# Differential Expression of Genes and DNA Methylation associated with Prenatal Protein Undernutrition by Albumen Removal in an avian model

**DOI:** 10.1038/srep20837

**Published:** 2016-02-10

**Authors:** Els Willems, Carlos Guerrero-Bosagna, Eddy Decuypere, Steven Janssens, Johan Buyse, Nadine Buys, Per Jensen, Nadia Everaert

**Affiliations:** 1 KU Leuven, Department of Biosystems, Laboratory of Livestock Physiology, Kasteelpark Arenberg 30 box 2456, 3001 Leuven, Belgium; 2 Linköping University, IFM Biology, AVIAN Behavioural Genomics and Physiology Group, Linköping 581 83, Sweden; 3 KU Leuven, Department of Biosystems, Research Group Livestock Genetics, Kasteelpark Arenberg 30 box 2456, 3001 Leuven, Belgium; 4 University of Liège, Gembloux Agro-Bio Tech, Precision Livestock and Nutrition Unit, Passage des Déportés 2, 5030 Gembloux, Belgium

## Abstract

Previously, long-term effects on body weight and reproductive performance have been demonstrated in the chicken model of prenatal protein undernutrition by albumen removal. Introduction of such persistent alterations in phenotype suggests stable changes in gene expression. Therefore, a genome-wide screening of the hepatic transcriptome by RNA-Seq was performed in adult hens. The albumen-deprived hens were created by partial removal of the albumen from eggs and replacement with saline early during embryonic development. Results were compared to sham-manipulated hens and non-manipulated hens. Grouping of the differentially expressed (DE) genes according to biological functions revealed the involvement of processes such as ‘embryonic and organismal development’ and ‘reproductive system development and function’. Molecular pathways that were altered were ‘amino acid metabolism’, ‘carbohydrate metabolism’ and ‘protein synthesis’. Three key central genes interacting with many DE genes were identified: UBC, NR3C1, and ELAVL1. The DNA methylation of 9 DE genes and 3 key central genes was examined by MeDIP-qPCR. The DNA methylation of a fragment (UBC_3) of the UBC gene was increased in the albumen-deprived hens compared to the non-manipulated hens. In conclusion, these results demonstrated that prenatal protein undernutrition by albumen removal leads to long-term alterations of the hepatic transcriptome in the chicken.

*In utero* growth retardation in humans as inferred from low birth weight has repercussions on postnatal health and performance, as exemplified by the increased risk of adult degenerative diseases such as type 2 diabetes^1^,^2^. The maternal low protein rat model is one of the most extensively studied animal models of *in utero* growth restriction^3^ and findings similar to those in humans are observed. The chicken can be used as a unique avian model to study prenatal protein undernutrition^4^,^5^,^6^,^7^ by replacing a part of the albumen with saline. As albumen is the main source of protein for the developing embryo^8^, the net effect is prenatal protein undernutrition. Thus, in the chicken only strictly nutritional effects are involved, in contrast to mammalian models where maternal effects (e.g. hormonal effects) are implicated. Indeed, in mammalian models manipulation of the maternal diet influences both the maternal nutritional and hormonal status, thereby exerting both nutritional and hormonal effects on the offspring.

Previously, the effects of albumen removal in the chicken have been studied by other groups^4^,^9^ and our own group^5^,^6^,^7^,^10^,^11^. Although the one-day-old chick weight was not significantly decreased by albumen removal, the body weight was reduced during the juvenile phase^5^,^7^,^11^. At adult age however, the effect of the body weight depended on the posthatch environmental conditions. When kept in battery cages with limited possibility for exercise, catch-up growth was observed in the albumen-deprived hens^11^. However when kept in floor pens–a more competitive environment for feed, space and water–the body weight of the albumen-deprived hens remained lower throughout the entire experimental period (55 weeks of age)^7^. Irrespective of the body weight in adulthood, the reproductive performance was markedly diminished by embryonic albumen removal as reflected in the reduced number and weight of the eggs^7^,^11^. At 10 weeks of age, glucose intolerance was observed in the albumen-deprived hens. This difference however, disappeared in adulthood due to age-related loss in glucose tolerance of the hens^7^.

Induction of an altered phenotype that persists throughout the lifespan implies stable changes in gene expression which would result in altered activities of metabolic pathways^12^. In the low-protein-diet rat model, changes in both hepatic gene expression and DNA methylation have been reported. Six days after weaning, the peroxisome proliferator-activated receptor α (PPARα) expression is 10.5-fold higher and DNA methylation 20.6% lower, whereas expression of the glucocorticoid receptor (GR) is 200% higher and DNA methylation 22.8% lower^13^. Moreover, these changes persist at least until postnatal day 34^14^. Gong *et al.* reported an increase in gene expression in the rat of the Insulin-like growth factor 2 (IGF2) in the liver and an increase in DNA methylation of the regulatory region of IGF2 in the liver of male low-protein diet offspring at day 0^15^.

Therefore, the objective of the present study was to perform a genome-wide screening for differences in gene expression using RNA-Sequencing (RNA-Seq) in liver samples collected from adult laying hens and differentially expressed genes were grouped according to biological function to discover affected pathways. In addition, it was investigated whether the alterations in gene expression coincided with changes in DNA methylation, in order to examine the possibility of epigenetic mechanisms underlying the observed long-term programming effects.

## Results

### Physiological results

Detailed physiological results have been published previously^7^. In brief, body weight of the albumen-deprived hens was reduced throughout the entire experimental period (0–55 weeks). In addition, the abdominal fat weight was also reduced in the albumen-deprived hens as compared to the sham-manipulated hens. No differences in absolute or relative liver weight were observed. The reproductive capacity was diminished in the albumen-deprived hens as reflected in the reduced number of eggs and lower egg weight. The plasma triiodothyronine (T_3_) levels were increased in the albumen-deprived compared to the non-manipulated hens, but not the sham-manipulated hens. An oral glucose tolerance test (OGTT) at 10 weeks of age revealed a decreased glucose tolerance in the albumen-deprived hens. During adulthood, an age-related loss of glucose tolerance was observed in the hens, leading to disappearance of treatment differences in the OGTT.

### Genome-wide screening for differentially expressed (DE) genes using RNA-Seq

**Figure 1:**
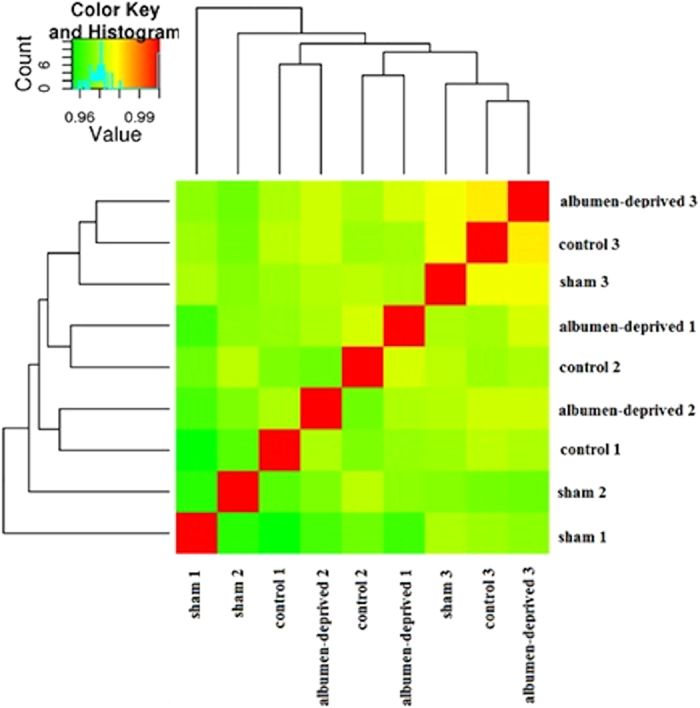
Correlation between biological replicates. Heat-map of Spearman’s correlation of the normalized counts as expression levels from all samples compared against each other, represented by a colored field ranging from green (0.95) to red (1).

**Figure 2:**
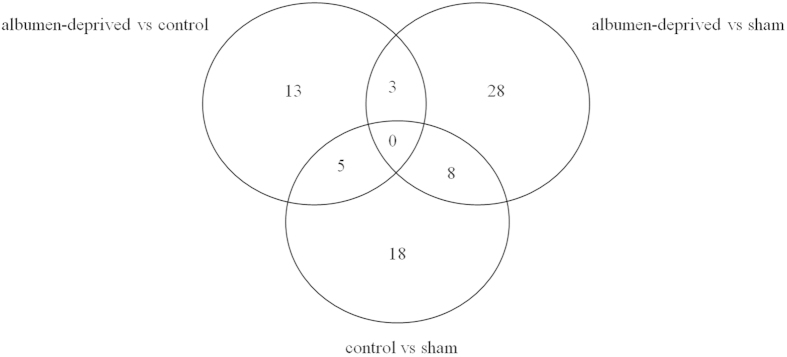
Venn-diagram showing 75 significantly differentially expressed (DE) genes. Genes are DE between the non-manipulated, sham-manipulated and albumen-deprived hens, including only previously identified genes. DE genes were filtered with a cut-off of P-value <0.001 and log_2_-fold change >1.

The logarithms of the fold change and associated P-values of treatment differences are depicted in supplementary Table S3 online. The heat map of Spearman correlation between all samples using the normalized counts as expression values is shown in Fig. 1. The correlation between all samples was very high (>0.95) and the biological replicates did not cluster well together. When using P < 0.001 and the log_2_-fold change higher than 1 as cut-off, 156 genes were differentially expressed between the treatments, only 75 of these were previously identified genes (Fig. 2). A heatmap generated based on these differentially expressed genes is depicted in Supplementary Figure 1 and demonstrates a good separation of the three treatments (except for sample sham 3).

Only 3 previously identified genes were differentially expressed between the albumen-deprived hens and both the non-manipulated and the sham-manipulated hens. To proceed with confirmation and validation, an additional 28 previously identified genes where the albumen-deprived hens differed from the sham-manipulated group were included in a list to select genes to validate the RNA-Seq, making a total of 31 genes (Table 1).

### Confirmation and validation of DE genes of RNA-Seq via qPCR

**Figure 3:**
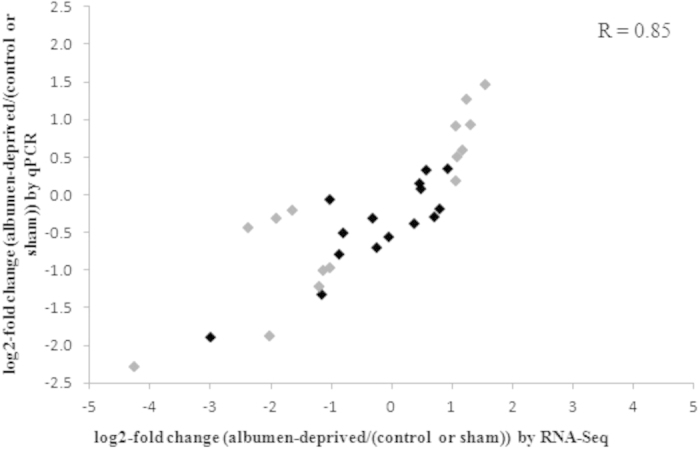
Correlation between RNA-Seq and qPCR results for 15 DE genes. The log_2_-fold change between the albumen-deprived hens and the non-manipulated (black box) and sham-manipulated hens (grey box) is displayed for both the RNA-Seq results as obtained from edgeR and the qPCR results obtained from the 2^−ddCt^ method. The Pearson’s correlation coefficient between relative expression levels is displayed.

Half of these 31 genes were selected (15 genes) for technical confirmation covering a range of expression levels and biological functions (Fig. 3). Relative expression levels of the albumen-deprived hens versus the non-manipulated and the sham-manipulated hens (n = 3 per group) are displayed as obtained from the RNA-Seq and qPCR results. The fold change estimates by qPCR and by RNA-Seq were strongly correlated (Pearson correlation coefficient was 0.85). The genes of which the expression levels did not fully match are the genes with low expression levels, pointing to decreased sensitivity of the RNA-Seq technique at low expression levels.

To validate the biological significance of the 15 DE genes, sample size was increased to 8 samples per group (Table 2). As observed from both the RNA-Seq and the qPCR results, 7 genes (46.7%) (TNFSF10, LAPTM4B, TMEM86A, CKS1B, NXPH-2, LRRC3C and BMF) had differentially expression in the liver of the albumen-deprived hens compared to the sham-manipulated hens (P < 0.1) and were thus validated. Eight genes could not be validated. Two genes of these (SEMA6D and H2B-I) had significantly increased expression in the albumen-deprived hens compared to the sham-manipulated hens in the RNA-Seq, but were significantly decreased in the qPCR results. Another 6 genes did not display significant differences in the gene expression measured by qPCR.

### Grouping of DE genes according to biological function

**Figure 4:**
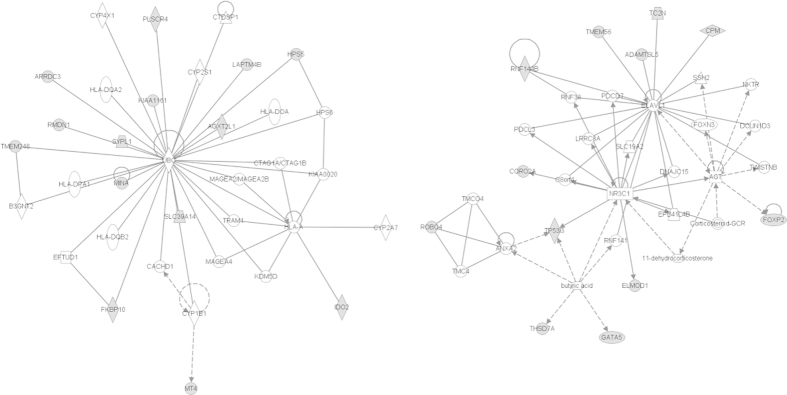
Grouping of DE genes according to biological function. Differentially expressed genes (P < 0.005 and fold change >1.5) are grouped with Ingenuity Pathway Analysis to identify affected pathways and key central regulatory genes. (**A**) Pathway involved in Embryonic development, organ development and organ morphology. (**B**) Pathway involved in Cell cycle and carbohydrate metabolism. Grey genes are identified as DE genes in RNA-Seq: 14 and 12 DE genes in the two pathways, respectively.

The cut-off criteria were loosened (P < 0.005 and Fold change >1.5) to include more DE genes in our analysis to find key metabolic and biological pathways affected by prenatal protein undernutrition by systems biology analysis using Ingenuity Pathways analysis (IPA) software. Only previously identified genes were included, and all DE genes for which the non-manipulated group differed from the sham-manipulated group were excluded as these do not represent effects of prenatal protein undernutrition. 116 DE genes were obtained, for 13 genes albumen-deprived differed from both non-manipulated and sham-manipulated; for 31 genes albumen-deprived differed from non-manipulated and for 88 genes albumen-deprived differed from sham-manipulated group. Significantly involved biological pathways are listed in Table 3. Two of these networks had key central genes, interacting with many DE genes. The first was a pathway involved in embryonic development, organ development and organ morphology, including 14 DE genes and had ubiquitin C (UBC) as central gene, whereas the second one was involved in cell cycle and carbohydrate metabolism, including 12 DE genes and had glucocorticoid receptor (NR3C1) and Embryonic lethal, abnormal Vision, Drosophila-like 1 (ELAVL1) as central genes (Fig. 4). None of the three central genes (UBC, NR3C1 and ELAVL1) were differentially expressed in the RNA-Seq dataset.

### DNA methylation analysis using MeDIP-qPCR

**Figure 5:**
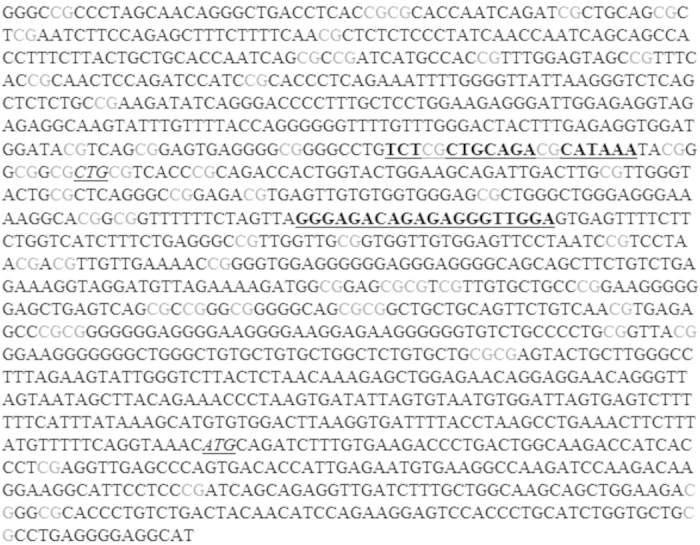
Part of the promoter sequence of UBC and the primer sequence of UBC_3 (depicted bold and underlined). The qPCR fragment is located around the transcription start site (CTG, italic underlined), starting from 31 bp upstream of the 5′-UTR (Untranslated Region) to 155 bp into this region and 553 bp upstream of the translation start codon (ATG, italic underlined). In the PCR fragment of the UBC_3 primers there are 14 CpG sites, of which one or more have differential methylation.

The 9 DE genes of the qPCR (TNFSF10, LAPTM4B, TMEM86A, CKS1B, NXPH-2, LRRC3C, BMF, SEMA6D and H2B-I) were selected for DNA methylation analysis. However, no specific primers for any of the CpG rich fragments of TNFSF10 could be optimised and this gene was therefore excluded from the analysis. In addition, the DNA methylation of the 3 key central regulatory genes (UBC, NR3C1, ELAVL1) identified by the pathway analysis was also examined. For each gene, several CpG rich fragments were examined in the promoter region or around the transcription start site via MeDIP-qPCR (Table 4). The DNA methylation of most of the examined fragments did not display a treatment effect. Only the DNA methylation of fragment (UBC_3) of the UBC gene was affected by treatment (P = 0.0442). The albumen-deprived hens had significantly more DNA methylation in this fragment than the non-manipulated group, whereas the sham-manipulated group had an intermediate value (Fig. 5).

## Discussion

The aim of the present study was to identify differences in gene expression using RNA-Seq and find pathways long-term affected by prenatal protein undernutrition by albumen removal in the liver of the chicken. In addition, it was investigated whether alterations in gene expression coincide with changes in DNA methylation (MeDIP-qPCR).

A first striking observation of the RNA-Seq dataset was the very high correlation between normalized counts as expression levels between all samples (Spearman correlation >0.95), as demonstrated by the heat map in Fig. 1. This shows that both within as well as between treatments’ differences in expression were very small and all samples were very similar. Indeed, posthatch environmental conditions were the same for all groups and therefore differences should only be due to the nutritional programming performed during embryonic development. A possible explanation for the small differences is that the treatments were applied early during embryonic development and measurements were performed in adulthood. The time span between treatment and sampling was nearly 58 weeks. Hence, treatment differences might have been much larger if measurements would have been performed earlier in life. However, at hatch, 2-D DIGE results revealed only 8 differential protein spots between the albumen-deprived hens and either the non-manipulated or the sham-manipulated hens or both^5^. Perhaps, other organs than the liver such as the hypothalamus are the sites of major changes in gene expression causing the phenotypic differences. Indeed, the hypothalamus functions as the center of regulation of energy and feed intake and is therefore a good candidate for future research.

Using P < 0.001 and log_2_-fold change >1 as cut-off to determine DE genes in RNA-Seq analysis, 3 genes were DE between the albumen-deprived and both the non-manipulated and sham-manipulated group, 39 between the albumen-deprived and the sham-manipulated and 21 between the albumen-deprived and the non-manipulated group (Fig. 2). To our knowledge, no published studies have examined the effect of low protein diet in mammalian models on the offspring via RNA-Seq. Previously, the effect of low protein diet during gestation on the offspring has been analyzed by microarray in the liver of mouse^16^ and rat^17^. In adult rat offspring (day 84), only a small number of genes was affected, 222 genes were upregulated, whereas 89 were downregulated (False discovery rate <0.05; fold change ≥1.5)^17^, although this number was much larger than in the present study. This could point at a distinction between application of strictly nutritional effects and involvement of secondary maternal effects. In addition, in these mammalian models the reduction in protein content in the diet is frequently accompanied by an increase in carbohydrates (e.g. glucose, sucrose or starch) as the diets need to be isocaloric. Therefore, observed effects might just as well be effects of carbohydrate overload instead of protein restriction^18^. Technical arguments to explain the low number of DE genes include lack of annotation of certain genes in the chicken. Indeed, from the present dataset 156 genes were differentially expressed, but only 75 of these were annotated in the databank. Although many of these ‘novel genes’ are probably mapping artefacts, some of these might represent true new genes. RNA-Seq datasets will become very important in the near future to improve the annotation of the chicken genome and identify more genes.

Expression of 15 DE genes of the RNA-Seq dataset was validated using qPCR. Technically (n = 3), a strong correlation between results from both techniques could be seen. The genes of which the expression levels did not fully match are the genes with low expression levels, pointing to decreased sensitivity of the RNA-Seq technique at low expression levels. Biologically (n = 8), however, only half of the DE genes showed the same results using qPCR. This is in agreement however with the level of consistency observed in other studies^19^. The differences between both techniques may be ascribed to several factors such as interindividual variation as liver samples were collected from different chickens. Many publications of genome-wide expression studies lack validation, which may provide misleading conclusions.

DE genes (P < 0.005 and fold change >1.5) were grouped to find key metabolic and biological pathways affected by prenatal protein undernutrition using systems biology analysis (Ingenuity Pathways analysis (IPA) software). Seven important physiological system development and functions were affected by the applied treatment. These pathways include ‘embryonic and organismal development’, ‘organ morphology and development’, ‘tissue development’ and ‘reproductive system development and function’, as expected by applying a treatment early during embryonic development. These results are in agreement with our previously published data on both the peri- and postnatal development of the albumen-deprived hens^5^,^6^,^7^,^11^. Although no differences were detected in one-day-old chick weight, the proportional carcass weight and the water content of the carcass were increased in the albumen-deprived group^5^. In addition, on embryonic day 20, the plasma thyroxine (T_4_) concentration was reduced for the albumen-deprived group, indicating a decreased metabolic rate. Body weight and feed intake were reduced during the young to juvenile phase, whereas at adult age the body weight either decreased or increased depending on the posthatch environmental conditions^7^,^11^. These results are probably the consequence of differences in embryonic development. Still, it is remarkable that this effect was still apparent at adult age. In agreement, maternal protein restriction in the rat offspring affected the ‘developmental process’ as biological pathway process^17^.

Genes involved in the ‘reproductive system development and function’ pathway were also demonstrated to be affected. Irrespective of posthatch body weight, the reproductive performance of the albumen-deprived hens was seriously diminished as reflected in both a reduced number and weight of the eggs^7^,^11^. Moreover, these eggs had a different composition (increased proportional yolk and decreased proportional albumen) and were of inferior quality as more second grade eggs were laid by the albumen-deprived group^11^. The reduced reproductive performance is in agreement with the study of Rae *et al.* linking prenatal undernutrition of ewes to diminished ovulation rate of offspring in adult life^20^.

Additionally, several molecular and cellular functions were affected: ‘amino acid metabolism’, ‘molecular transport’, ‘small molecule biochemistry’, ‘cell death and survival’, ‘cell-to-cell signaling and interaction’, ‘carbohydrate metabolism’ and ‘protein synthesis’. Lillycrop *et al.* also found altered molecular functions due to prenatal protein undernutrition in rats: receptor binding, tetrapyrrole binding, and UDP-glycosyltransferase activity, cation and anion transmembrane transporter activity, growth factor activity and ATPase activity^17^. Genes with a wide range of functions were demonstrated to be altered in the present study, which is consistent with our previous studies demonstrating a wide range of both physiological and metabolic alterations. By partial albumen replacement with saline, the embryonic protein availability was decreased and therefore changes in the expression of genes related to the amino acid metabolism could be expected. Moreover, changes in amino acid metabolism were also observed by screening for differential protein abundances in the liver of newly hatched chicks. Affected pathways included valine, methionine, glutamate and cysteine degradation^5^, although the precise genes/proteins affected differed between these two studies. In this respect, changes in protein synthesis were also inferred. A previous study from our group also found indications of an altered protein metabolism in broilers treated by albumen removal before incubation, suggesting a transient increase in muscle proteolysis^10^. Although these results may be interpreted as ordinary and expected, it provides evidence that the applied treatment creates strictly nutritional changes.

Another affected pathway was the ‘carbohydrate metabolism’, although few genes were represented here. However, changes in glucose metabolism have been demonstrated before by differential protein abundances in the liver of newly hatched chicks. Affected pathways included the gluconeogenesis, the tricarboxyl acid cycle and pyruvate fermentation to lactate^5^. Furthermore, the albumen-deprived hens exhibited reduced glucose tolerance at 10 weeks of age possibly by a decreased insulin production or increased insulin resistance. However this difference disappeared at adult age due to age-related loss of glucose tolerance in the chicken^7^. Also, in mammalian maternal low protein models, differences in gene expression involved in the carbohydrate metabolism were observed. In rat dams, fed a protein-restricted diet, an increase of the regulatory enzyme of the gluconeogenesis phosphoenolpyruvate carboxykinase PCK1 mRNA and increased activity was detected in liver of the progeny until 11 months of age, suggesting that programming of the metabolism also extends to the regulation of gene expression^21^.

In offspring from low-protein-diet rat models, alterations in DNA methylation were observed in association with changes in gene expression^13^,^14^. In the present study, a screening of the DNA methylation of the 9 DE genes and the three key central genes (UBC, NR3C1 and ELAVL1) was performed by MeDIP-qPCR. The data suggest that DNA methylation of fragment UBC_3 of the ubiquitin C (UBC) gene is increased in the albumen-deprived hens compared to the non-manipulated hens. The fact that the gene expression in this region did not differ in the RNA-Seq experiment does not exclude the possibility that these changes in DNA methylation could be related with distal regulation of other genes. The fragment is located around the transcription start site, starting from 31 bp upstream of the 5′-UTR (Untranslated Region) to 155 bp into this region and 553 bp upstream of the translation start codon (CTG) (Fig. 5). 14 CpG’s are contained within this fragment and one or more of these CpGs have increased methylation in the albumen-deprived hens. Ubiquitin C is one of the genes that produces ubiquitin, the precursor of polyubiquitin. Ubiquination is associated with protein degradation, DNA repair, cell cycle regulation, kinase modification, endocytosis, and regulation of other cell signaling pathways. Since the present study only performed a DNA methylation analysis biased towards specific genes, a genome-wide approach will be needed in order to determine the real extent of changes in DNA methylation that could be generated as result of the experimental treatment used. Moreover, other epigenetic modifiers such as histone modification or microRNA could be examined, as demonstrated in a low protein rat model^22^,^23^.

## Conclusions

In conclusion, the present results demonstrate for the first time that prenatal protein undernutrition by albumen removal leads to long-term alterations of the hepatic transcriptome in the chicken. As expected, pathways involved in embryonic development were affected by this treatment. In addition, changes in amino acid metabolism and protein synthesis prove the efficacy of the application of strictly nutritional effects. Limited effects of DNA methylation were observed in the regulation of the currently examined DE genes.

## Methods

### Ethics statement

All experiments were conducted in strict accordance with the European Communities Council Directive (2010/63/EC) and were approved by the Institutional Ethical Committee of KU Leuven (P002–2012).

### Chickens

The set-up of this experiment was previously described by^7^. More information on the technique of albumen removal is available in^11^. In brief, fertilized Isa Brown layer-type eggs (Vepymo, Poppel, Belgium) were randomly divided over the three treatments and incubated together. After one day of incubation, a hole was drilled in the egg, 3 ml of outer thin albumen was removed using a needle and syringe and replaced by about the same volume of sterile saline, followed by sealing of the hole using a drop of paraffin (albumen-deprived group). A sham-manipulated group was mock-treated similar to the albumen-deprived group, except for the actual albumen removal and saline injection. A third group received no treatment (non-manipulated group). A discussion of the technique of albumen removal can be found in^10^,^11^. After hatch, only the female chicks were reared in floor pens with wood shavings as litter until 55 weeks of age. All floor pens were located in one environmentally controlled room. Room temperature was initially set at 34 °C and was gradually decreased until 20 °C at 5 weeks of age. This temperature was maintained until 55 weeks of age. A 23 h light cycle was initially provided and gradually decreased to 10 h at 6 weeks. At 14 weeks, the light cycle was gradually prolonged to 15 h at 19 weeks to stimulate sexual maturation. The hens received soy-wheat-corn based diets *ad libitum* formulated according to the developmental requirements based on the Isa Brown Management Guide (Research Diet Services, Wijk bij Duurstede, the Netherlands)^11^. At 55 weeks of age, the laying hens were killed and 8 liver samples per group were randomly collected and snap frozen in liquid nitrogen before storage at −80 °C. Frozen liver tissue was homogenized by grinding into powder in liquid nitrogen before use.

### Genome-wide screening for differential gene expression using RNA-Seq

#### RNA isolation

Total RNA was extracted from approximately 50 mg of crushed liver collected from hens at 55 weeks of age (n = 8 per group) as previously described by^5^. The RNA concentration and quality (260/280 ratio) was measured using UV-spectroscopy (Implen, Westburg, Leusden, The Netherlands). The integrity of the RNA was electrophoretically verified. In addition, integrity of RNA samples used for RNA-Seq was checked on the BioAnalyzer (Agilent Technologies, Santa Clara, CA, USA).

#### Sequencing and quality control of the reads

The sequencing was performed in collaboration with the Genomics Core of the UZ Leuven (Belgium). Samples (n = 3 per group, 2 μg RNA) were prepared by TruSeq library preparation (RS122, Illumina, San Diego, CA, USA) to generate single end unstranded sequencing libraries, according to the manufacturer’s recommendations. Sequencing of all samples was carried out in 1 lane of 1 flow cell on the HiSeq2000 (Illumina), using single end chemistry with read lengths of 50 base pairs. Between 17.3 and 26.5 million reads were sequenced for each sample. The quality of the reads was checked using the FASTQC software (Illumina). All the reads passed the Phred score (≥28).

#### Data analysis of the RNA-Seq data

Data analysis was performed in collaboration with the Nucleomics Core of the VIB (Belgium). Reads were mapped to the chicken genome (Galgal4.71) using TopHat (v2.0.8b)^24^. Quality filtering was performed with SAMtools (0.1.19–44428cd) removing all reads from the alignment that are non-primary mappings or have a mapping quality ≤ 20^25^. The number of mapped reads varied between 16 and 24.8 million reads per sample as calculated by Picard (1.92) (BroadInstitute) resulting in a mapping efficiency of approximately 93%. Per sample, the transcripts were identified from the mapping with Cufflinks v2.1.1^26^. With the Cuffmerge tool, all per-sample transcript lists were merged together with the reference annotation into one file. With Cuffcompare, the identified transcripts were matched with known transcripts and genes, based on overlapping coordinates on the reference genome. About 22.1% novel loci were identified, resulting in a total of 22,082 genes. The number of reads in the alignments that overlap with the gene features were counted using HTSeq-count (0.5.4p3)^27^. The reads that could be attributed to more than one gene (ambiguous, about 1.3 million per sample) or could not be attributed to any gene (no feature, about 1.1 million per sample) were not counted. Exclusion caused by ambiguity excluded about 6.9% and no feature about 6.1% of the mapped reads per sample. The 8,483 genes that had less than 1 counts-per-million were considered ‘absent’ and therefore removed from the dataset^28^. As such, 13,599 genes were left. Per sample, GC-content was corrected using full quantile normalization on bins of GC-content with the EDASeq package from Bioconductor^29^ for within-sample normalization. Between-sample normalization was carried out to correct for library size and RNA composition, as it is known that these are sources of large variation, using full quantile normalization with the EDASeq package from Bioconductor^30^. The statistical modeling assumed the design of the experiment as log (Count) = β_1_ × non-manipulated + β_2_ × sham-manipulated + β_3_ albumen-deprived. For each gene, the coefficients β were estimated with the EdgeR package (3.2.3)^31^ of Bioconductor, by fitting a negative binomial generalized linear model (GLM)^28^. The following comparisons were tested: non-manipulated vs. albumen-deprived, sham-manipulated vs. albumen-deprived and sham-manipulated vs. non-manipulated. Differentially expressed genes were selected for confirmation by qPCR based on the criteria that the p-value should be < 0.001 combined with a cut-off fold change of 2 or absolute log_2_-ratio larger than 1^32^.

### Identification of relevant biological pathways

A list of differentially expressed genes for pathway analysis was created by selecting the differentially expressed transcripts with P < 0.005 and fold change >1.5. This list of differentially expressed genes between the albumen-deprived hens and both non-manipulated and sham-manipulated hens or only the sham-manipulated hens was imported into the Ingenuity Pathway Analysis (IPA; Ingenuity Systems, Redwood City, CA, USA) to identify biological interactions between the gene products. The biological interaction scores were defined by the IPA statistical algorithm based on its knowledge base, and the P value was calculated according to Fisher’s exact test.

### Confirmation and validation of RNA-Seq results via qPCR

#### DNase treatment and reverse transcription

RNA samples (n = 8 per group) were DNase treated with RQ1 RNase-Free DNase (M6101, Promega, Fitchburg, WI, USA), according to the manufacturer’s instructions. The RNA was transcribed into cDNA using the Reverse Transcription system (A3500, Promega, Madison, WI, USA). Denaturation was performed for 3 min at 80 °C followed by 45 min at 42 °C for the reverse transcription. The reaction was terminated by heating the samples for 5 min at 95 °C.

#### Primer design

The primer design was described previously^5^. In supplementary Table S1 online, all primers are listed, both for reference genes and the genes of interest. All measurements were performed in the linear range of amplification with correlation coefficient >0.99. Final primer concentration was 0.3 μM for all primer pairs, except for BMF, where 0.15 μM was used.

#### qRT-PCR

Quantitative real-time PCR (qRT-PCR) measurements were performed as described previously^5^. For the analysis of the qRT-PCR output, the 2^−ΔΔCT^ method of relative quantification was used^33^. Expression of genes was normalized to the geometric average of the two references genes: β-actin (ACTB) and peptidylprolylisomerase D (PPID).Technical confirmation results were based on the same three samples as used in the RNA-Seq, whereas in the biological validation the sample size was increased to 8 samples per group.

### Methylated DNA immunoprecipitation and quantitative real-time PCR (MeDIP-qPCR)

#### Genomic DNA isolation

One ml of SNET buffer (1% SDS; 0.4M NaCl; 5 mM EDTA; 20 mM Tris.HCl pH 8.0) and 20 μl of Proteinase K (00120437, Thermo Scientific, Waltham, MA, USA) was added to 100 mg of homogenized liver tissue and incubated overnight in a water bath at 56 °C. The next day 1 ml of a phenol: chloroform: isoamyl alcohol 25:24:1 mixture (A0889, AppliChem, Darmstadt, Germany) was added and thoroughly vortexed. After centrifugation (14,000 rpm, 10 min, 4 °C), the top layer was transferred to a new tube and the process of adding, vortexing and centrifugating was repeated several times with addition of subsequently 1 ml of the phenol: chloroform: isoamyl alcohol 25:24:1 mixture and 1 ml of chloroform (1.02445, Merck Millipore, Readington Township, NJ, USA). Finally, 1 ml of isopropanol (20842, Prolabo, VWR, West Chester, PA, USA) was gently mixed with the top layer, centrifuged (14,000 rpm, 5 min, 4 °C) and the resulting supernatant was discarded. The DNA pellet was washed with 500 μl 70 ethanol (14,000 rpm, 5 min, 4 °C) and the pellet was dissolved in 150 μl milliQ water. The DNA concentration and purity was measured on a NanoDrop 1000 Spectrophotometer (Thermo Scientific, Waltham, MA, USA).

#### Shearing of genomic DNA

6 μg of genomic DNA was diluted in 30 μl milliQ and subsequently fragmented by sonication in icecold water in the Biorupter (Diagenode, Liège, Belgium) for 1 to 4 cycles of 15 times 10 sec ON–10 sec OFF (and briefly spinned down after each cycle) until the fragment size was between 100–600 bp, with the majority of the fragments around 250 bp. Fragment size was verified by electrophoresis on a 2% agarose gel (A9539, Sigma-Aldrich, St. Louis, MO, USA).

#### Methylated DNA immunoprecipitation (MeDIP)

This method was previously described by^34^. 5.5 μg of fragmented genomic DNA was diluted to 400 μl in TE buffer (10 mM Tris.HCl pH 7.5, 1 mM EDTA) and denatured for 10 minutes at 95 °C, followed by immediate cooling on ice for 5 minutes. 100 μl of 5X IP buffer (50 mM Na.H_2_PO_4_ pH 7, 0.7M NaCl, 0.4 mM Triton X-100) and 2.5 μl 5-mC monoclonal antibody (C15200006, Diagenode) were added and incubated overnight on a rotating platform at 4 °C. Protein A/G PLUS-Agarose Immunoprecipitation Reagent (sc-2003, Santa Cruz Biotechnology, Inc, Dallas, TX, USA) was pre-washed with 0.1 mg/ml BSA in 1X PBS and resuspended in 40 μl IP buffer. Beads were added to the DNA-antibody complex and incubated for 2 h on a rotating platform at 4 °C. Beads bound to the DNA-antibody complex were washed 3 times with 1 ml 1X IP buffer. To release the methylated DNA from the beads, the beads were resuspended in 250 μl digestion buffer (50 mM Tris.HCl pH 8, 10 mM EDTA, 0.5% SDS), 3.5 μl proteinase K (00120437, Thermo Scientific) and incubated overnight on a rotating platform at 55 °C. The methylated DNA was purified by first mixing with 250 μl phenol solution (P4557, Sigma-Aldrich) and centrifugation (14,000 rpm, 5 min, room temperature) and repeating this with addition of 250 μl chloroform:isoamyl alcohol (24:1, C0549, Sigma-Aldrich). Washing was performed by addition of 2 μl GlycoBlue Coprecipitant (AM9515, Applied Biosystems, Foster City, CA, USA), 20 μl 5M NaCl and 500 μl 99.5% icecold ethanol and placement at −20 °C for at least 1 hour. After centrifugation (14,000 rpm, 15 min, 4 °C), the supernatant was removed and the pellet was washed with 500 μl of icecold 70% ethanol and centrifuged again. The pellet was resuspended in 30 μl milliQ. The DNA concentration was measured on the NanoDrop 1000 Spectrophotometer (Thermo Scientific). The yield was about 10–30 ng/μl.

#### Whole Genome amplification

30 ng of each MeDIP sample was amplified using the GenomePlex Complete Whole Genome Amplification kit (WGA2, Sigma-Aldrich) according the manufacturer’s instruction, except no fragmentation was performed, as the samples were already sonicated. After amplification, the DNA was cleaned using the GeneJET PCR Purification kit (K0701, Thermo Scientific). The DNA concentration was measured on the NanoDrop 1000 Spectrophotometer (Thermo Scientific) and the samples were diluted to 20 ng/μl.

#### Selection of CpG rich regions around transcription start site of interesting genes and primer design

For each gene of interest, several CpG rich (>4 CpG’s) fragments (150–200 bp) were selected in the promoter region (5000 bp upstream of transcription start site) and around the transcription start site and the corresponding primers for these fragments were designed (supplementary Table S2 online) using Primer Designing Tool (NCBI). The primers were purchased from Life Technologies (Carlsbad, CA, USA) and verified as described previously.

#### Quantitative real-time PCR (qPCR)

Quantitative real-time PCR (qRT-PCR) measurements were performed in triplicate using the LightCycler480 qPCR machine (Roche Applied Science, Penzberg, Germany). For every reaction, 5 μl of LightCycler 480 SYBR Green I Master Mix (04 887 352 001, Roche), 0.5–1 μM of forward and reverse primer, 2.5 μl of DNA (20 ng/μl) and milliQ to a final volume of 10 μl were added together in a 96-well plate (LightCycler 480 96 Multiwell Plate white, 04 729 692 001, Roche) and sealed with LightCycler 480 Sealing Foil (04 729 757 001, Roche). The PCR reaction program began with 5 min heating at 95 °C followed by 45 cycles of 10 sec at 95 °C, 20 s at 60 °C and 30 sec at 72 °C. In addition, a melting curve analysis was performed to check the specificity of the primers (5 seconds at 95 °C, 1 minute at 55–60 °C, temperature gradually increased until 97 °C is reached).

### Statistical Analysis

qPCR and MeDIP-qPCR data were processed with the statistical software package SAS version 9.3 (SAS Institute Inc., Cary). The gene expression values (2^−ddCt^) and Ct values of the MeDIP-qPCR were analyzed using one-way ANOVA with treatment (non-manipulated, sham-manipulated and albumen-deprived) as independent variable. When a significant effect of treatment was demonstrated, the means were further compared by a posthoc Tukey’s test. All data are shown as mean ± SEM.

## Additional Information

**Data availability**: The data sets supporting the results of this article are available in the NCBI's Gene Expression Omnibus repository35, and are available through GEO Series accession number GSE57938 (http://www.ncbi.nlm.nih.gov/geo/query/acc.cgi?acc=GSE57938).

**How to cite this article**: Willems, E. *et al.* Differential Expression of Genes and DNA Methylation associated with Prenatal Protein Undernutrition by Albumen Removal in an avian model. *Sci. Rep.* **6**, 20837; doi: 10.1038/srep20837 (2016).

## Supplementary Material

Supplementary Information

Supplementary Table S3

## Figures and Tables

**Table 1:** List of differentially expressed genes from RNA-Seq.

	Gene	Description	Accession	log_2_-fold change			P value		
				C vs. A	S vs. A	C vs. S	C vs. A	S vs. A	C vs. S
**C≠A; S≠A**	GRIN2C	glutamate receptor	ENSGALG00000027415	3.98	3.53	0.45	0.00018	0.00065	NS
	IDO2	indoleamine 2,3-dioxygenase2	ENSGALG00000024085	3.91	3.22	0.69	0.00001	0.00020	NS
	*SPAG-4 like*	sperm-associated antigen 4-like	ENSGALG00000000443	−3.03	−4.28	1.25	0.00021	0.00000	NS
**S≠A**	*H2B-I*	histone H2B 1/2/3/4/6	ENSGALG00000027174	0.35	1.04	−0.69	NS	0.00059	NS
		uncharacterized protein	ENSGALG00000008635	1.01	2.51	−1.49	NS	0.00001	NS
	H2A-VII	histone H2A-IV	ENSGALG00000027113	0.66	1.06	−0.41	NS	0.00034	NS
	*SEMA6D*	semaphorin 6D	ENSGALG00000004844	0.69	1.07	−0.38	NS	0.00012	NS
	*HERPUD1*	homocysteine-inducible, endoplasmic reticulum stress-inducible, ubiquitin-like domain member 1	ENSGALG00000001220	−0.34	−1.21	0.87	NS	0.00003	NS
	IP6K2	inositol hexakisphosphate kinase 2	ENSGALG00000005701	0.41	1.07	−0.66	NS	0.00092	NS
	*SLC6A6*	sodium- and chloride-dependent taurine transporter	ENSGALG00000006425	0.76	1.16	−0.40	NS	0.00060	NS
	PROK2	prokineticin 2	ENSGALG00000007785	−0.91	−2.93	2.02	NS	0.00083	NS
	*LECT2*	myeloid protein 1 precursor	ENSGALG00000006323	−1.17	−2.03	0.87	NS	0.00038	NS
	TMEM116	transmembrane protein 116	ENSGALG00000004760	0.40	1.03	−0.63	NS	0.00052	NS
	*LAPTM4B*	lysosomal protein transmembrane 4 beta	ENSGALG00000028628	−0.90	−1.92	1.02	NS	0.00071	NS
	GATA5	transcription factor GATA-5	ENSGALG00000005352	2.55	3.42	−0.87	NS	0.00012	NS
			ENSGALG00000000850	−0.89	−1.83	0.94	NS	0.00066	NS
	*CKS1B*	CDC28 protein kinase regulatory subunit 1B	ENSGALG00000028664	−1.04	−1.65	0.61	NS	0.00047	NS
	*LRRC3C*	leucine rich repeat containing 3C	ENSGALG00000026789	−0.83	−2.39	1.56	NS	0.00073	NS
		uncharacterized protein	ENSGALG00000010993	0.67	1.46	−0.79	NS	0.00007	NS
	GAL7	gallinacin-7	ENSGALG00000022817	−0.92	−1.87	0.95	NS	0.00071	NS
	GAL2	gallinacin-2	ENSGALG00000016669	−1.36	−2.47	1.10	NS	0.00004	NS
	*GJA1*	gap junction alpha-1 protein	ENSGALG00000014873	−0.27	−1.15	0.87	NS	0.00025	NS
	SLITRK4	SLIT and NTRK-like family, member 4	ENSGALG00000007242	1.31	4.45	−3.14	NS	0.00009	NS
	*TMEM86A*	transmembrane protein 86A	ENSGALG00000006358	0.91	1.29	−0.38	NS	0.00078	NS
		uncharacterized protein	ENSGALG00000009387	−1.08	−2.26	1.18	NS	0.00038	NS
	*BMF*	bcl-2-modifying factor	ENSGALG00000014537	0.46	1.04	−0.58	NS	0.00002	NS
	TC2N	tandem C2 domains, nuclear	ENSGALG00000010738	1.04	1.37	−0.32	NS	0.00043	NS
	*NXPH2*	neurexophilin 2	ENSGALG00000029083	0.55	1.54	−0.99	NS	0.00009	NS
	*GLUL*	glutamine synthetase	ENSGALG00000003678	−0.08	−1.04	0.96	NS	0.00061	NS
	*TNFSF10*	tumor necrosis factor ligand superfamily member 10	ENSGALG00000009179	0.44	1.23	−0.80	NS	0.00064	NS
	HMCN1	hemicentin 1	ENSGALG00000005141	−0.57	−1.49	0.92	NS	0.00017	NS

Cut-off criteria were P < 0.001 and log_2_-fold change > 1 between the albumen-deprived hens and the non-manipulated and sham-manipulated hens (3 genes) or between the albumen-deprived hens and the sham-manipulated hens (28 genes). Genes in italic were selected for measurements of gene expression via qPCR.

**Table 2:** Biological validation of RNA-Seq results.

	Gene	qPCR (2^−ddCt^)			P-value	Validated?
		non-manipulated	sham-manipulated	albumen-deprived		
**C≠A; S≠A**	SPAG4like	1.00 ± 0.27	0.56 ± 0.23	0.75 ± 0.17	NS	No
**S≠A**	TNFSF10	1.00 ± 0.13^b^	0.69 ± 0.09^b^	1.40 ± 0.07^a^	0.0006	Yes
	LAPTM4B	1.00 ± 0.16^ab^	1.96 ± 0.62^b^	0.50 ± 0.09^a^	0.0520	Yes
	TMEM86A	1.00 ± 0.07^ab^	0.93 ± 0.15^b^	1.42 ± 0.16^a^	0.0489	Yes
	CKS1B	1.00 ± 0.12^ab^	1.74 ± 0.39^a^	0.96 ± 0.08^b^	0.0777	Yes
	NXPH2	1.00 ± 0.08^a^	0.45 ± 0.07^b^	1.06 ± 0.20^a^	0.0091	Yes
	LRRC3C	1.00 ± 0.14^ab^	1.68 ± 0.45^a^	0.70 ± 0.10^b^	0.0776	Yes
	BMF	1.00 ± 0.10^ab^	0.74 ± 0.08^b^	1.32 ± 0.12^a^	0.0048	Yes
	SEMA6D	1.00 ± 0.17^ab^	1.70 ± 0.50^a^	0.63 ± 0.08^b^	0.0916	No
	H2B-I	1.00 ± 0.16^ab^	2.12 ± 0.68^a^	0.67 ± 0.08^b^	0.0681	No
	GLUL	1.00 ± 0.14	1.20 ± 0.23	1.13 ± 0.12	NS	No
	HERPUD1	1.00 ± 0.10	1.38 ± 0.36	1.17 ± 0.29	NS	No
	SLC6A6	1.00 ± 0.11	1.18 ± 0.21	1.03 ± 0.06	NS	No
	LECT2	1.00 ± 0.29	1.33 ± 0.33	0.65 ± 0.16	NS	No
	GJA1	1.00 ± 0.11	0.94 ± 0.11	0.97 ± 0.12	NS	No

Gene expression of selected DE genes (P < 0.001 and log_2_-fold change >1) were measured in 8 samples per treatment via qPCR. ^a,b^treatment means with different superscript are significantly different (P < 0.1).

**Table 3:** Identification of relevant biological pathways affected by prenatal protein undernutrition by albumen removal in the chicken.

Molecular and cellular functions	Molecules	P-value	Number of molecules
Amino acid metabolism	SLC3A1, SLC7A10, ASNS, DIO2, GFPT2, IDO2, SLC6A6	2.91E-05–4.78E-02	7
Molecular transport	SLC3A1, SLC7A10, TNFSF10, MTTP, LRAT, SLC6A6, DCT, IP6K2, RACGAP1, SLC20A2, SLC39A14, ABCC9, GRIN2C, SIK1, DIO2, CD200, NR0B1, ULK1, FOXP2, HPS5	2.91E-05–4.78E-02	20
Small molecule biochemistry	SLC3A1, SLC7A10, ASNS, DIO2, GFPT2, IDO2, SLC6A6, TP53I3, ABP1, LRAT, MTTP, PDE11A, TNFSF10, DCT, FOXP2, HPS5	2.91E-05–4.78E-02	16
Cell death and survival	DCT, PRF1, TNFSF10, PITX2, CD200, LAMA2, BMF, SIK1, LECT2, SLC6A6, PROK2, IP6K2, FRZB	4.11E-04–4.78E-02	13
Cell-to-cell signaling and interaction	DCT, PRF1, TNFSF10, FRZB, BAIAP2, LAMA2, CD200, PITX2, HPS5,TECTA, FOXP2, IL12RB1	3.48E-03–4.78E-02	12
Carbohydrate metabolism	TNFSF10, GFPT2	5.42E-03–4.26E-02	2
Protein synthesis	MTTP, CD200, GRIN2C, NR0B1, ULK1, IL12RB1, PRF1	5.42E-03–2.28E-02	7
**Physiological system development and function**			
Nervous system development and function	CD200, LAMA2, FOXP2, BAIAP2, PDE11A, ARHGEF28, SLITRK4, ULK1, FRZB, PRF1, TECTA,	4.30E-04–4.78E-02	11
Organ morphology	LRAT, NR0B1, ADAMTS1, ERRFI1, GATA5, LAMA2, FRZB, PITX2, SLC39A14, SLC6A6, TECTA, FOXP2, WNT11, DCT, HPS5, ABCC9, PLCL1, CD200, PDE11A, DIO2	4.30E-04–4.86E-02	20
Reproductive system development and function	LRAT, NR0B1, ADAMTS1, ERRFI1, GATA5, LAMA2, BMF, WNT11, PROK2, PRF1, TNFSF10, PITX2	4.30E-04–3.74E-02	12
Tissue development	CD200, LAMA2, ADAMTS1, WNT11, NR0B1, FOXP2, BMF, DIO2, FRZB, PITX2, ERRFI1, SLC39A14, PROK2, HPS5, ARHGEF28, BAIAP2, SLITRK4, ULK1, LRAT, TNFSF10, ADAMTS5	4.30E-04–4.78E-02	21
Embryonic development	WNT11, LAMA2, NR0B1, FOXP2, PITX2, ADAMTS1, FRZB, SLC39A14, BMF, DIO2, CKS1B, PROK2, ERRFI1, HPS5, LRAT	5.42E-03–4.78E-02	15
Organ development	WNT11, NR0B1, FOXP2, DIO2, ADAMTS1, PITX2, FRZB, SLC39A14, BMF, PROK2, ERRFI1, HPS5, LECT2, MTTP, PDE11A, PRF1, TNFSF10, LAMA2, LRAT	5.42E-03–4.78E-02	19
Organismal development	ADAMTS1, BMF, NR0B1, PITX, DIO2, FRZB, ERRFI1, FOXP2, SLC39A14, GATA5, HPS5, LAMA2, LRAT, WNT11, PROK2, TNFSF10	5.42E-03–4.78E-02	16

Differential expressed genes were grouped through the use of Ingenuity Pathway Analysis (IPA). IPA-analysis (www.ingenuity.com) was used to identify key biological pathways comprising the differentially identified proteins after prenatal protein undernutrition by albumen removal in chicken. The significance of the canonical pathways was tested by Fisher’s exact test. The following genes are included in the biological pathways. Abbreviations: ABCC9 (ABC transporter C family member 9); ABP1 (Actin binding protein 1); ADAMTS1 and 5 (A disintegrin and metalloproteinase with thrombospondin motifs 1 and 5); ARHGEF28 (Rho guanine nucleotide exchange factor 28); ASNS (Asparagine synthetase); BAIAP2 (Brain-specific angiogenesis inhibitor 1-associated protein 2); BMF (Bcl-2-modifying factor); CD200 (OX-2 membrane glycoprotein); CKS1B (CDC28 protein kinase regulatory subunit 1B): DCT (L-dopachrome tautomerase precursor); DIO2 (Iodothyronine deiodinase); ERRFI1 (ERBB receptor feedback inhibitor 1); FOXP2 (Forkhead box protein P2); FRZB (secreted frizzled-related protein 3 precursor); GATA5 (transcription factor GATA-5); GFPT2 (glutamine-fructose-6-phosphate transaminase 2); GRIN2C (glutamate receptor); HPS5 (Hermansky-Pudlak syndrome 5); IDO2 (indoleamine 2,3-dioxygenase 2); IL12RB1 (interleukin 12 receptor, beta 1); IP6K2 (inositol hexakisphosphate kinase 2); LAMA2 (laminin, alpha 2); LECT2 (myeloid protein 1 precursor); LRAT (Lecithin retinol acyltransferase); MTTP (microsomal triglyceride transfer protein large subunit precursor); NR0B1 (nuclear receptor subfamily 0 group B member 1); PDE11A (phosphodiesterase 11A); PITX2 (pituitary homeobox 2); PLCL1 (phospholipase C-like 1); PRF1 (Perforin-1); PROK2 (prokineticin 2); RACGAP1 (Rac GTPase activating protein 1); SIK1 (serine/threonine-protein kinase); SLC20A2 (sodium-dependent phosphate transporter 2); SLC39A14 (solute carrier family 39 (zinc transporter), member 14); SLC3A1 (Neutral and basic amino acid transport protein); SLC6A6 (sodium- and chloride-dependent taurine transporter); SLC7A10 (Asc-type amino acid transporter 1); SLITRK4 (SLIT and NTRK-like protein 4); TECTA (tectorin alpha); TNFSF10 (tumor necrosis factor ligand superfamily member 10); TP53I3 (tumor protein p53 inducible protein 3); ULK1 (Serine/threonine-protein kinase); WNT11 (Protein Wnt-11).

**Table 4:** DNA methylation analysis of genes of interest.

Gene	Accession number	Primer pair	MeDIP-qPCR (normalized Ct values)			
			non-manipulated	sham-manipulated	albumen-deprived	P-value
BMF	ENSGALG00000014537	BMF_1	1.00 ± 0.02	1.03 ± 0.02	1.03 ± 0.01	NS
CKS1B	ENSGALG00000028664	CKS1B_1	1.00 ± 0.01	1.03 ± 0.02	1.00 ± 0.00	NS
H2B-I	ENSGALG00000027174	H2B_1	1.00 ± 0.04	1.00 ± 0.04	0.96 ± 0.05	NS
		H2B_2	1.00 ± 0.01	1.02 ± 0.00	1.01 ± 0.01	NS
		H2B_3	1.00 ± 0.02	1.08 ± 0.05	1.01 ± 0.02	NS
LAPTM4B	ENSGALG00000028628	LAPTM4B_1	1.00 ± 0.02	1.03 ± 0.02	1.00 ± 0.02	NS
		LAPTM4B_2	1.00 ± 0.03	1.06 ± 0.02	0.99 ± 0.02	NS
		LAPTM4B_3	1.00 ± 0.02	0.99 ± 0.02	1.00 ± 0.03	NS
LRRC3C	ENSGALG00000026789	LRRC3C_1	1.00 ± 0.02	0.99 ± 0.02	0.95 ± 0.03	NS
		LRRC3C_2	1.00 ± 0.02	1.05 ± 0.05	1.02 ± 0.04	NS
		LRRC3C_3	1.00 ± 0.01	1.02 ± 0.03	1.00 ± 0.02	NS
NXPH2	ENSGALG00000029083	NXPH2_1	1.00 ± 0.04	0.99 ± 0.04	0.95 ± 0.03	NS
		NXPH2_2	1.00 ± 0.01	1.00 ± 0.02	1.00 ± 0.01	NS
		NXPH2_3	1.00 ± 0.04	0.96 ± 0.03	0.94 ± 0.03	NS
SEMA6D	ENSGALG00000004844	SEMA6D_1	1.00 ± 0.01	0.99 ± 0.01	0.98 ± 0.01	NS
		SEMA6D_2	1.00 ± 0.01	0.99 ± 0.01	0.99 ± 0.01	NS
TMEM86A	ENSGALG00000006358	TMEM86A_1	1.00 ± 0.02	0.97 ± 0.01	1.00 ± 0.02	NS
ELAVL1	ENSGALG00000000726	ELAVL1_1	1.00 ± 0.01	1.00 ± 0.01	0.99 ± 0.01	NS
		ELAVL1_2	1.00 ± 0.01	1.00 ± 0.01	0.99 ± 0.01	NS
		ELAVL1_3	1.00 ± 0.01	0.99 ± 0.01	0.99 ± 0.01	NS
NR3C1	ENSGALG00000007394	NR3C1_1	1.00 ± 0.03	1.00 ± 0.03	0.96 ± 0.02	NS
		NR3C1_2	1.00 ± 0.02	1.01 ± 0.03	0.98 ± 0.02	NS
		NR3C1_3	1.00 ± 0.01	1.00 ± 0.01	1.00 ± 0.01	NS
		NR3C1_4	1.00 ± 0.04	0.99 ± 0.05	0.95 ± 0.04	NS
UBC	ENSGALG00000004509	UBC_1	1.00 ± 0.03	0.97 ± 0.01	1.00 ± 0.02	NS
		UBC_2	1.00 ± 0.01	1.00 ± 0.01	0.99 ± 0.01	NS
		UBC_3	1.00 ± 0.00^b^	1.02 ± 0.01^ab^	1.03 ± 0.01^a^	0.0442

Analysis was performed for 9 DE genes from RNA-Seq and qPCR and 3 key central genes identified by pathway analysis by MeDIP-qPCR.
