# Supplementary material for: Differential Expression of Genes and DNA Methylation associated with Prenatal Protein Undernutrition by Albumen Removal in an avian model
Source: Sci Rep. 2016 Feb 10;6:20837. doi: 10.1038/srep20837 (PMC4748411; doi:10.1038/srep20837)
Supplement: Supplementary Information [file srep20837-s1.pdf]

Differential Expression of Genes and DNA Methylation associated with Prenatal Protein Undernutrition by Albumen Removal in an avian model

Els Willems[1,2], Carlos Guerrero-Bosagna[2], Eddy Decuypere[1], Steven Janssens[3], Johan Buyse[1], Nadine Buys[3], Per Jensen[2], Nadia Everaert[1,4]

Supplementary Table S1: **List of primers used for quantification of the gene expression via qPCR.** The selected genes had a differentially expression in the RNA-Seq results where expression in the liver of the albumen-deprived hens differed from the sham-manipulated group (14 genes) or in addition from the non-manipulated group (1 gene). ACTB and PPID were reference genes used for normalization.

Gene	Accession number (Ensembl)	Forward 5'-primer-3'	Reverse 5'-primer-3'	Size (bp)
BMF	ENSGALG00000014537	CTCCACATACAGCGGCATCA	AGCTGCCACCACACTTGATT	50
CKS1B	ENSGALG00000028664	CCAAAGACATCGCCAAGCTG	GGAGGATGTGTGGCTCTGG	129
GJA1	ENSGALG00000014873	CAGCAGCGCCAATATCCAGT	AAGAGGACAGACAGCCACAC	113
GLUL	ENSGALG00000003678	TATGGTGTCCAACCAGCACC	GCAGTAGTACGGACCTTGGG	124
H2B-I	ENSGALG00000027174	AACGACATCTTCGAGCGCAT	TGTACTTGGTGACCGCCTTG	166
HERPUD1	ENSGALG00000001220	CTGGACCATCAGCTCCTGC	TGAGCAGCTTTAACCTCCGT	122
LAPTM4B	ENSGALG00000028628	CGCGTATAAGCAACATGCGG	CTGGCGAAGGTAGTCCTGAA	130
LECT2	ENSGALG00000006323	TGCTCCCTTTTCTGGTGAGC	GACACAGAAGCCTGATCCCC	100
LRRC3C	ENSGALG00000026789	CGCCATGGTGATCTCCTACC	GGGCAGCGATTTGAGGTACT	88
NXPH2	ENSGALG00000029083	GCGTCCTCCGGATCGTATTT	ACAGGCGTAGTGGGTTGATG	138
SEMA6D	ENSGALG00000004844	AGGCAAGGCTGTGTATTCCC	AGAACGAATCCCCAGGAACG	134
SLC6A6	ENSGALG00000006425	TCTGGGAGCGCAATGTACTG	ACAGTGCCAGATCCCACTTG	75
SPAG4like	ENSGALG00000000443	CGCAGACCTTCTTGCTAAAGAATA	CACTCGGTAAAGGCAGGTGT	107
TMEM86A	ENSGALG00000006358	GGCTACTTCATTCATGGTCTGC	TCATGCCAAAGGCTGAGGAG	70
TNFSF10	ENSGALG00000009179	GAAGTGGCACCTGGGAAAGT	CCTGTCACCGTTGATTGCAG	82
ACTB	ENSGALG00000009621	TCGCCCCAGACATCAGGGTGTGA	TTGCTCTGGGCTTCATCACCAACGT	75
PPID	ENSGALG00000009451	GTCGCACCCGTCCCCTGTAGA	ATTCGTCCAACTCGCTCTCCCC	93

For every gene the Ensembl accession number, the sequences of the forward (F) and reverse primer (R) and the size of the amplified fragment (bp) are provided.

Supplementary Table S2: **List of primers used for quantification of the DNA methylation of the genes of interest.** Genes were selected based on differential expression both in RNA-Seq and qPCR (9 genes). In addition, 3 key central genes of pathways affected by albumen removal were also included.

Gene	Accession number	Primer pair	Forward 5'-primer-3'	Reverse 5'-primer-3'	Ta (°C)	Primer conc. (µM)	Size (bp)	# CpG
BMF	ENSGALG00000014537	BMF_1	GCACGGAGGAATCCCATTGA	CCTACAGCTCGCTCCAGTTC	58	0.75	150	19
CKS1B	ENSGALG00000028664	CKS1B_1	TGTGATGCTGCCCAAAGACA	GGGACGTAGCACAGTGTCAG	60	0.5	199	10
H2B-I	ENSGALG00000027174	H2B_1	CCCCTTCGGCGTCTTACAG	CCGTTTGGTATTCAGCCCCT	55	0.75	197	24
		H2B_2	TCCAACCAACAAACCAACCG	TGGAAGTTCGTGTTTGTGGC	58	0.75	199	28
		H2B_3	CAAGAAGAGCCGCAAGGAGA	ATGGTCGAGCGCTTGTTGTA	60	0.5	180	19
LAPTM4B	ENSGALG00000028628	LAPTM4B_1	GTCTGGTACCTGGTAAGCGG	GCGTTAAAGGGAGCAGCAAG	60	0.5	184	17
		LAPTM4B_2	GGATTCTTCGGTCTCGTGGG	GAGCAGAGGATCAGCACCAG	60	0.5	161	13
		LAPTM4B_3	TGAGCTCCTGTGCTTCCTTG	GCTCGGGGTGAAGATGTGAA	60	1	185	11
LRRC3C	ENSGALG00000026789	LRRC3C_1	GCTCAACCCTCGGCTTACTT	TGCCGTAACTCGGTGCAG	60	0.5	198	22
		LRRC3C_2	CGAGGTCCCCAAAGACATCC	GGAGGACAGGTCCAGGGAG	60	0.5	199	18
		LRRC3C_3	GACGTCCTCAGCAACGTGAA	ACGTTGCAGAAGTCCGTGTC	60	0.5	200	19
NXPH2	ENSGALG00000029083	NXPH2_1	AAATCGCGTTCTGTGGGATG	TTCGACACGACGTTACACCT	60	0.5	191	25
		NXPH2_2	AGGTGTAACGTCGTGTCGAA	CCTTCTCACGCCGTAAGGTT	60	0.5	196	13
		NXPH2_3	TGGGTTCGTTTCAAGGGGAG	GCCGCTACGAGTTTTCACCA	60	0.5	191	23
SEMA6D	ENSGALG00000004844	SEMA6D_1	GTGCCAGTGCTCAAATGCAA	ATTAGCATCACAGAGGCGCA	60	0.5	188	6
		SEMA6D_2	GGCTGGCAATCATCAGCTTC	TTGCATTTGAGCACTGGCAC	60	0.5	178	5
TMEM86A	ENSGALG00000006358	TMEM86A_1	GGGGAGTCGCGAAGCTTAAT	GAAACAGGAGCATGCGAAGC	60	0.5	197	22
ELAVL1	ENSGALG00000000726	ELAVL1_1	ATACATCAGTGGGCTCCCGA	AGGTGCAGTGCATCACAGTC	60	0.5	154	9
		ELAVL1_2	TCTGAAGGAAACGCTCCGAC	TCTGCCGATGTCATCTCTGC	60	0.5	168	7
		ELAVL1_3	ACGTGAAGTCAGAAAGGCCC	CCAACCACAAACACGCTCAG	60	0.5	170	9
NR3C1	ENSGALG00000007394	NR3C1_1	TGAACAAGTCCTCGAGCCTG	TCCAGGGTGCTTTGGTCTTC	60	0.5	184	16
		NR3C1_2	CTCAACAGGTCCCGACACAT	GAACCGCTCCATCTGACGAC	55	0.75	176	30
		NR3C1_3	GTTATTGCTGCGGTTTCCCC	GGAACGAGATCAGCTCAGGG	60	0.5	200	26
		NR3C1_4	TCTGATCGCTCCTGCTCTCT	TCACTGCGAGGTGATTTGGG	60	0.5	200	18
UBC	ENSGALG00000004509	UBC_1	TGTGATGCTGTGGTTACGGG	GAGAGGGCAGATTTCGGTGG	58	0.75	196	9
		UBC_2	CTAGCAACAGGGCTGACCTC	GGATCTGGAGTTGCGGTGAA	58	0.5	183	11
		UBC_3	TCTCGCTGCAGACGCATAAA	TCCAACCCTCTCTGTCTCCC	60	0.5	186	14

For every gene the Ensembl accession number, the sequences, the annealing temperature (Ta, °C) and primer concentration (µM) of the forward and reverse primer pair, the size of the amplified fragment (bp) and the number of CpG in this fragment (# CpG) are provided.

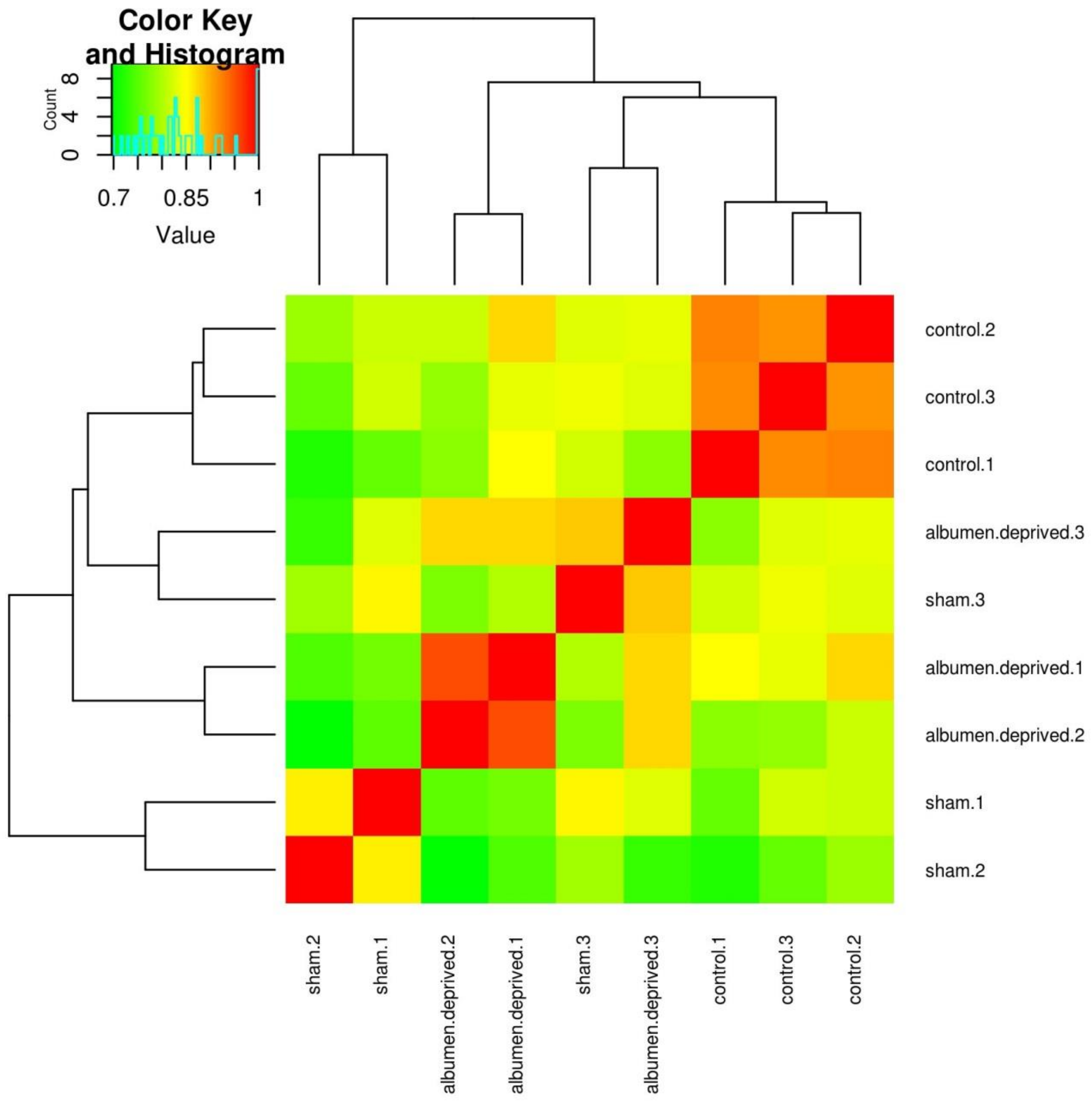

Supplementary Figure S1: Correlation between biological replicates based on the 75 differentially expressed genes ($P < 0.001$ and log2 FC > 1). Heat-map of Spearman's correlation of the normalized counts as expression levels from all samples compared against each other, represented by a colored field ranging from green (0.95) to red (1).
